# Severe pancreatitis complicated by abdominal compartment syndrome managed with decompressive laparotomy: a case report

**DOI:** 10.1186/s12893-019-0575-8

**Published:** 2019-08-17

**Authors:** Adele Hwee Hong Lee, Wen-Shen Lee, David Anderson

**Affiliations:** 1 Austin Hospital, Melbourne, Victoria 3084 Australia; 2 The Alfred Hospital, Melbourne, Victoria 3004 Australia; 3 Department of Intensive Care, The Alfred Hospital, Melbourne, Victoria 3004 Australia

**Keywords:** Case reports, Intra-abdominal hypertension, Pancreatitis, Laparotomy, Decompression, surgical, Multiple organ failure

## Abstract

**Background:**

Abdominal compartment syndrome (ACS) is an uncommon complication of severe pancreatitis. It is defined as a sustained intraabdominal pressure (IAP) > 20 mmHg (with or without an abdominal perfusion pressure (APP) < 60 mmHg), associated with new organ dysfunction/failure. ACS confers a poor prognosis and should be promptly diagnosed and managed. However, it is often missed on clinical examination, leading to a delay of diagnosis.

**Case presentation:**

A 38-year old Sri Lankan man presented with severe alcohol-induced pancreatitis associated with abdominal compartment syndrome. Diagnosis was delayed due to false reassurance from clinical examination. The patient was managed with a decompressive laparotomy, after which he required treatment with continuous renal replacement therapy (CRRT), total parenteral nutrition (TPN) and broad-spectrum antibiotics for a prolonged period of time. Despite significant post-operative multi-organ failure, the patient survived.

**Conclusions:**

Early trans-bladder measurement of IAP is important for severe pancreatitis. Serial measurements of IAP should be implemented early when any known risk factor for ACS is present in a critically ill patient.

## Background

Although pancreatitis is often encountered in critically ill patients, abdominal compartment syndrome (ACS) as a complication is rarely encountered. It occurs in at least 15% of patients with severe pancreatitis, with a mortality rate of up to 49% [1, 2]. To diagnose ACS, a trans-bladder measurement of intra-abdominal pressure (IAP) is required [3]. It is vital for diagnosis to be made early so management can be expedited. The principles of management involve treating reversible aetiologies, trial of non-operative and percutaneous interventions for pancreatitis, before escalating to surgical decompression if appropriate. Recent evidence has shown improved outcomes in selected patients when surgical decompression was performed early [4–8].

**Fig. 1:**
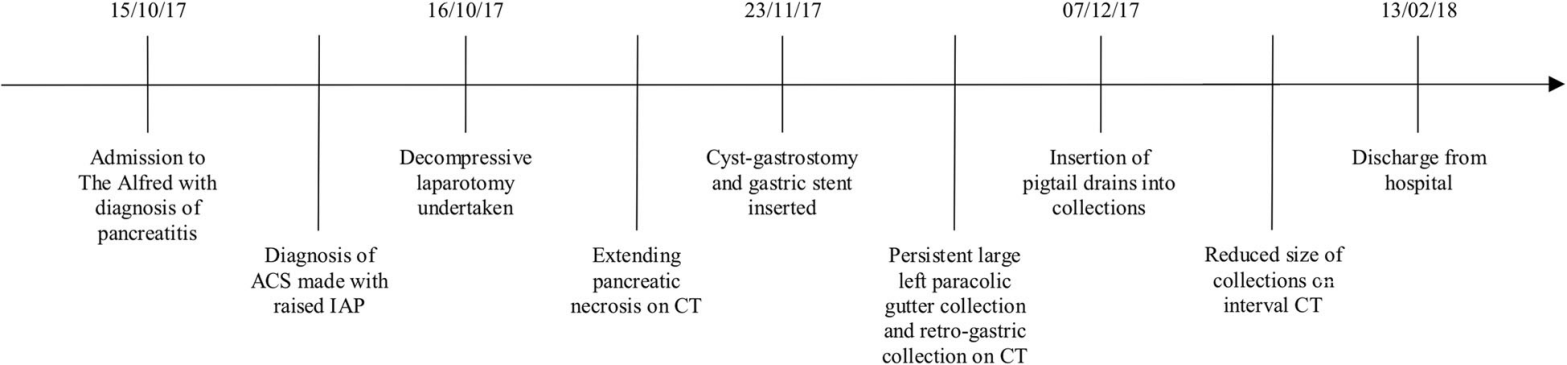
Timeline summarising important clinical events. ACS: Abdominal compartment syndrome; CT: computed tomography; IAP: intraabdominal pressure

A case of severe pancreatitis complicated by ACS, induced by alcohol, is presented. This occurred between October 2017 and February 2018 (Fig. 1). A brief discussion of the incidence, risk factors, pathophysiology and management is also presented.

## Case presentation

A 38-year-old Sri Lankan man was brought in by ambulance to the Emergency Department (ED) following a one-day history of severe abdominal pain on a background of chronic heavy alcohol intake and a recent binge of 500 ml of spirits three days prior. He described the pain as constant and radiating to the left shoulder, with associated severe vomiting. His past medical history included chronic liver disease secondary to his alcohol abuse, with no other known complications of chronic alcoholism such as cardiomyopathy or neuropathy reported. Other comorbidities included smoking, schizophrenia, obesity and previous intravenous drug use.

**Fig. 2:**
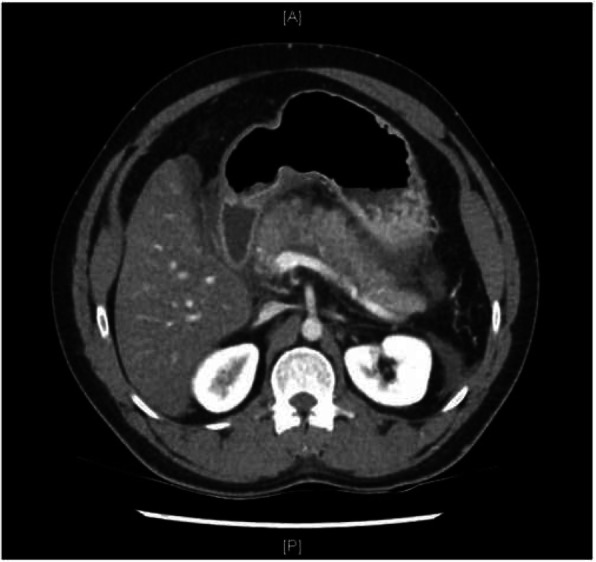
Computed tomography of the abdomen performed on presentation demonstrating findings consistent with uncomplicated acute pancreatitis. Axial view

On arrival, the patient had a Glasgow Coma Scale (GCS) of 15. He was afebrile and hemodynamically stable with a blood pressure of 140/95, heart rate of 90 and saturating at 94% on room air. His abdomen was overtly distended but did not feel tense. Generalised tenderness was elicited on palpation with no signs of peritonism. Acute pancreatitis was confirmed with an elevated serum lipase of 7111. He also had raised white cell count of 16, elevated lactate of 2.9, elevated hematocrit of 0.54, deranged liver function tests (ALT 222, ALP 124, GGT 457) and elevated creatinine of 124. An abdominal computerised tomography (CT) scan revealed findings consistent with uncomplicated acute pancreatitis with no evidence of ascites (Fig. 2). The impression was that of alcohol-induced acute pancreatitis associated with acute kidney injury (AKI) and hepatic dysfunction. He was managed with analgesia, anti-emetics and intravenous fluids.

Over the following few hours, the patient’s condition deteriorated. Pain remained severe despite high doses of opioids. The patient looked pale and diaphoretic with early signs of respiratory distress. The patient was afebrile and normotensive but persistently tachycardic at 150 beats per minute despite aggressive fluid resuscitation with compound sodium lactate (6 l over 24 h). This was thought to be appropriate given his haemoglobin of 200. His lactate climbed to 3.3 and potassium to 5.5. His hematocrit then was 0.56. He was then transferred to the Intensive Care Unit (ICU) for ongoing fluid resuscitation and pain management, with a modified Glasgow-Imrie criteria of six points. During this time, the patient’s abdomen examined to be distended and firm but not tense.

Over the next hour, the patient desaturated with increased work of breathing and agitation. The decision was made to intubate him. Rapid-sequence induction was performed with low dose fentanyl, ketamine and full dose rocuronium. Following induction, the patient became bradycardic and subsequently developed pulseless electrical activity (PEA). He was not hypoxaemic immediately prior to the arrest. Return of spontaneous circulation was achieved after 5 min with 3 rounds of cardiopulmonary resuscitation (CPR) and administration of 1 mg adrenaline, 0.6 mg of atropine and 1 L of 0.9% normal saline.

Post-intubation, the patient became progressively difficult to ventilate with very poor pulmonary compliance. Tidal volume was less than 200 ml on a driving pressure of 15 and a PEEP of 15. PaO_2_ remained in the 70s despite FiO2 of 1.0. Arterial blood gas (ABG) analysis showed the following: pH 6.90, pCO_2_120 mm Hg, pO_2_ 74 mmHg and HCO_3_ 23 mEq/L. The high ventilator requirements did not correlate with findings of a mobile chest x-ray – mild bibasal collapse and oedema without evidence of acute respiratory distress syndrome (ARDS). He was unresponsive to fluid challenge and required an infusion of 45 mcg/min of noradrenaline and 2 mcg/min of vasopressin in order to maintain an adequate mean arterial pressure (MAP). Bedside echocardiogram did not show signs of hypovolemic, cardiogenic or obstructive shock. The patient was thought to be developing AKI, being oliguric (< 10 ml/h) with a potassium of 6.5.

Intra-abdominal pressure measured via the bladder was 28 mmHg. The patient was initially planned for diuretic therapy instead of a decompressive laparotomy, given the high risk of hypovolaemia, fistulation and ventral hernia formation. However, he was deemed impossible to ventilate with worsening pulmonary compliance, and diuresis with frusemide was thought to be futile in achieving a negative fluid balance due to high pressor requirements. The decision was then made for an emergency decompressive laparotomy. Immediately upon opening the abdominal cavity, the pulmonary compliance improved and the vasopressor requirement fell dramatically. Respiratory acidemia resolved rapidly with a pH of 7.39 and pCO_2_ of 45 mmHg. About one litre of ascitic fluid was drained and omental adhesions were divided. Other findings included severe retroperitoneal oedema and an oedematous pancreas, with no signs of organ necrosis. The laparotomy was closed with a vacuum-assisted closure (VAC) dressing in situ with planned dressing changes every three days. Primary closure was not used to avoid undue accumulation of intrabdominal pressure and to facilitate premeditated ‘re-looks’ and drainage required.

Day two post-laparotomy, the patient was weaned off noradrenaline with a MAP stable at 100 mmHg. He was commenced on CRRT. Nasogastric feeding was commenced. The patient was extubated two weeks post-laparotomy.

**Fig. 3:**
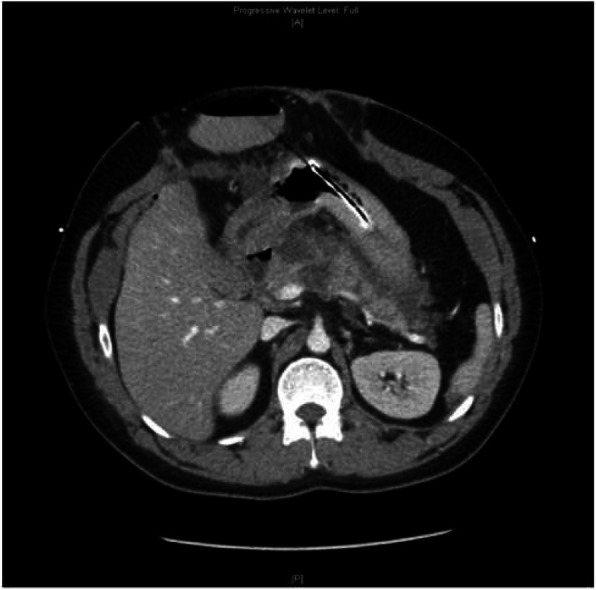
Computed tomography of the abdomen a week after surgical decompression revealed heterogeneous enhancement in the pancreatic head and body consistent with necrosis. An immature collection is starting to form adjacent to the pancreatic tail. Axial view

**Fig. 4:**
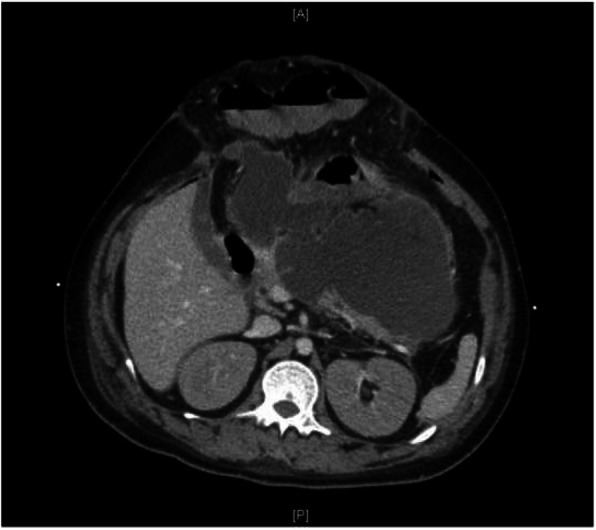
Computed tomography of the abdomen a month after surgical decompression revealed a large retroperitoneal collection seen within lesser sac between pancreatic tail and stomach, consistent with walled-off necrosis. Axial view

Post-operatively, the patient’s pancreatitis was complicated by an extending pancreatic necrosis with an acute necrotic collection (Fig. 3). The patient was also persistently febrile and tachycardic. Aspergillus Fumigutus complex was identified on sputum cultures and Proprionibacterum Acnes was identified on blood cultures. The decision was made to commence meropenem and teicoplanin, with adjuncts of fluconazole and ivermectin. These were later ceased with subsequent negative cultures and clinical improvement. A month later, a repeat CT revealed that the necrosis had stabilised with the formation of a walled-off necrosis (Fig. 4). This was subsequently drained via endoscopic ultrasound-guided cyst-gastrostomy and stent insertion. A retro-gastric collection and a left para-colic collection found were also drained.

A week after stent insertion, the patient was deemed stable enough to be transferred to the ward. The fascial layer was subsequently closed with nylon sutures, a total of two and a half months after the first operation, and skin was closed with polypropylene sutures 4 days after. Repeat CT showed reduction in size of the walled-off necrosis with no new collections seen.

The patient underwent a successful recovery and was discharged to subacute care after 44 days in ICU and 50 days on the ward. He spent a total of 121 days in hospital. A follow-up by telephone 2 months later revealed no further issues and that the patient was satisfied with the outcome.

## Discussion and conclusions

Severe acute necrotising pancreatitis is defined as pancreatitis associated with persistent organ failure (i.e. > 48 h), in our case persistent acute kidney dysfunction [9]. It occurs in up to 20% of patients and is associated with mortality rates of 8 to 40% [1–10]. It is a known risk factor for ACS. ACS can develop in at least 15% of patients with severe pancreatitis [2], and is associated with a higher mortality rate than patients without ACS (49% vs 11%) [1].

As of the guidelines by the World Society of the Abdominal Compartment Syndrome (WSACS) published in 2013, abdominal compartment syndrome (ACS) is defined as a sustained intraabdominal pressure (IAP) > 20 mmHg (with or without an abdominal perfusion pressure (APP) < 60 mmHg) which is associated with new onset organ failure. It can be divided into primary and secondary, where primary ACS is associated with injury or disease in the abdominopelvic region while secondary ACS refers to those that are not. The pathogenesis of ACS is multifactorial, developing from a combination of diminished abdominal wall compliance, increased intra-abdominal contents, increased intra-luminal contents, capillary leakage, fluid resuscitation as well as age, bacteraemia, shock and sepsis. In pancreatitis, this revolves around pancreatic inflammation causing pancreatic and visceral oedema with surrounding fluid collections, as well iatrogenic processes such as aggressive fluid resuscitation. Ascites from capillary leak, paralytic ileus and gastric distension by duodenal obstruction raises IAP [1]. This ultimately leads to hypoperfusion and ischaemia of abdominal viscera.

This is a case of primary ACS secondary to alcohol-induced acute pancreatitis. In addition to the aforementioned pathophysiological processes, the patient was obese with underlying hepatomegaly and hepatic dysfunction which would have further predisposed him to ACS.

Several case reports have described ACS secondary to pancreatitis. Most recently, Mora-Guzman et al. described a case of ACS secondary to acute necrotising pancreatitis. Despite initial percutaneous drainage of the necrotic collection, ACS developed with CT scan showing diffuse pancreatic necrosis, multiple collections with extensive haemorrhage associated with gastric and liver compression with visceral infarcts. A decompressive laparotomy was performed with haematoma evacuation and necrosectomy. Unfortunately, the patient died 20 days after presentation due to progression of pancreatitis and refractory multi-organ dysfunction. ACS was diagnosed seven days after presentation with severe pancreatitis when the patient deteriorated, as compared to the day of presentation in our case. The difference in outcomes was probably due to the established peripancreatic complications and advanced multi-organ dysfunction at the time of diagnosis [10].

Clinical examination for increased IAP is inaccurate. Hence, serial measurements of IAP should be implemented early when any known risk factor for ACS is present in a critically ill patient. Additionally, measurement of IAP can serve as a prognostic tool in pancreatitis [11]. Pupelis et al. found no mortality among patients who had IAP < 25 mmHg compared with 36% mortality in those with IAP > 25 mmHg [12]. Trans-bladder measurement is the most commonly used method of measuring IAP due to low cost and simplicity and is recommended as a first line investigation. In this case, measurement of IAP was delayed due to false reassurance from the patient’s non-tense abdomen, delaying the diagnosis and management of ACS [3].

ACS should be suspected when multi-organ dysfunction develops with increased intra-abdominal pressure. Oliguria is one of the hallmark signs of increased IAP as AKI develops due to decreased renal perfusion pressure from direct compression of renal arteries, veins and parenchyma, exacerbated by decreased cardiac output. Signs of AKI with hyperkalaemia developed early in our patient. He became hypoxic with increased ventilatory needs secondary to diaphragmatic splinting, with resultant increase in airway pressures and reduced pulmonary capacity. The PEA arrest post-induction was likely secondary to raised IAP impeding venous return, as well as autonomic dysfunction from fentanyl-induced sympatholysis, and abdominal distension causing increased vagal tone and an exaggerated parasympathetic response.

When ACS has been confirmed, reversible aetiologies should be promptly identified and treated. In the setting of pancreatitis, non-operative and percutaneous interventions should be trialled before escalation to surgical decompression. Such therapies include nasogastric decompression, prokinetics, sedation, neuromuscular blockers, diuretics or ultrafiltration, or percutaneous drainages of ascites. If such therapies are futile, surgical decompression should be implemented promptly, most commonly via a midline laparotomy [13]. The initial reluctance to proceed with surgical decompression after identification of ACS in our case could be explained by the fact that decompressive laparotomy has been shown to be associated with multiple complications and mortality of up to 50%, with variable recuperation of organ dysfunction even after decompression [14]. Most recently however, Jacob et al. reported no mortalities in five patients who received decompressive laparotomies for ACS with severe acute pancreatitis. Post-operatively there was a marked improvement in organ function and ventilatory effort. All survived and made a good recovery [4]. Overall, studies reporting improved outcomes with decompressive laparotomy recommend early intervention and hence this should be considered early in selected patients [5–8].

Post-operatively, non-operative management options should be undertaken to reduce IAP. Post-decompressive laparotomy, we performed serial scans on our patient to identify intra-abdominal lesions, where pancreatic necrosis associated with collections were identified and subsequently drained. Abdominal wall compliance was improved with adequate sedation, analgesia and neuromuscular blockade. We optimised fluid administration with the aim of zero to negative balance, assisted with CRRT. TPN was slowly introduced to minimise intraluminal contents. This is especially useful when the risk of a paralytic ileus is high. It is also recommended to measure IAP at least every four hours while the patient is critically ill. A decrease in frequency of IAP measurements can occur after IAP is consistently below 12 mmHg.

Administration of fluid therapy in the context of pancreatitis is an important concept to grasp in order to prevent ACS. It has the potential to precipitate ACS and can affect measurements of volume status such as central venous pressure and urine output. Initially, more fluid should be administered as patients with higher IAP tend to be intravascularly deplete with third-space losses. This has to be done with tight monitoring of IAP. If IAP increases or ACS develops, fluid administration should be slowed or immediate measures to reduce IAP should be implemented [5]. Mao et al. in 2010 reported that goal-directed fluid therapy of 5 to 10 ml/kg/h resulted in lower ACS incidence and mortality as opposed to aggressive fluid resuscitation, and thus recommended this approach in severe acute pancreatitis [15]. It is difficult to assess if aggressive fluid resuscitation in our case led to poorer outcomes given that it was clearly indicated due to tachycardia and hemoconcentration.

In this case report, we describe a case of ACS successfully managed with decompressive laparotomy. Its seemingly indolent presentation delayed diagnosis and subsequent treatment. Despite this and complications of multi-organ failure, the patient survived.

ACS confers a poor prognosis in patients with severe acute pancreatitis. A high index of suspicion is needed for prompt diagnosis and management. This case highlights the inaccuracy of clinical examination in detecting increased IAP and the importance of early trans-bladder measurement of IAP when a patient presents with severe pancreatitis. Serial measurements of IAP should be implemented early when any known risk factor for ACS is present in a critically ill patient. Decompressive laparotomy showed a satisfactory outcome and should be considered for ACS, however there is insufficient evidence to suggest its routine use. Higher quality studies assessing the treatment of ACS are warranted.

## Data Availability

All data generated or analysed during this study are included in this published article.

## Abbreviations

ABG: Arterial blood gas
ACS: Abdominal compartment syndrome
AKI: Acute kidney injury
APP: Abdominal perfusion pressure
ARDS: Acute respiratory distress syndrome
CPR: Cardiopulmonary resuscitation
CRRT: Continuous renal replacement therapy
CT: Computerised tomography
ED: Emergency department
GCS: Glasgow coma scale
IAP: Intraabdominal pressure
ICU: Intensive care unit
MAP: Mean arterial pressure
PEA: Pulseless electrical activity
TPN: Total parenteral nutrition
VAC: Vacuum-assisted closure
WSCAS: World Society of the Abdominal Compartment Syndrome
